# *In Vitro* Antibacterial Activity of Microbial Natural Products against Bacterial Pathogens of Veterinary and Zoonotic Relevance

**DOI:** 10.3390/antibiotics13020135

**Published:** 2024-01-30

**Authors:** Stefanie A. Barth, Daniel Preussger, Jana Pietschmann, Andrea T. Feßler, Martin Heller, Werner Herbst, Christiane Schnee, Stefan Schwarz, Florian Kloss, Christian Berens, Christian Menge

**Affiliations:** 1 Friedrich-Loeffler-Institut—Federal Research Institute for Animal Health (FLI), Institute of Molecular Pathogenesis, 07743 Jena, Germany; 2 Institute of Microbiology and Epizootics, Freie Universität Berlin, 14163 Berlin, Germany; 3 Veterinary Centre for Resistance Research (TZR), Freie Universität Berlin, 14163 Berlin, Germany; 4 Institute of Hygiene and Infectious Diseases of Animals, Justus-Liebig-University, 35392 Giessen, Germany; 5 Transfer Group Anti-Infectives, Leibniz Institute for Natural Product Research and Infection Biology, Leibniz-HKI, 07745 Jena, Germany

**Keywords:** natural products, antibacterial activity, antimicrobial susceptibility testing, *Mannheimia haemolytica*, *Pasteurella multocida*

## Abstract

Antimicrobial resistance (AMR) is considered one of the greatest threats to both human and animal health. Efforts to address AMR include implementing antimicrobial stewardship programs and introducing alternative treatment options. Nevertheless, effective treatment of infectious diseases caused by bacteria will still require the identification and development of new antimicrobial agents. Eight different natural products were tested for antimicrobial activity against seven pathogenic bacterial species (*Brachyspira* sp., *Chlamydia* sp., *Clostridioides* sp., *Mannheimia* sp., *Mycobacterium* sp., *Mycoplasma* sp., *Pasteurella* sp.). In a first pre-screening, most compounds (five out of eight) inhibited bacterial growth only at high concentrations, but three natural products (celastramycin A [CA], closthioamide [CT], maduranic acid [MA]) displayed activity at concentrations <2 µg/mL against *Pasteurella* sp. and two of them (CA and CT) also against *Mannheimia* sp. Those results were confirmed by testing a larger collection of isolates encompassing 64 *Pasteurella* and 56 *Mannheimia* field isolates originating from pigs or cattle, which yielded MIC_90_ values of 0.5, 0.5, and 2 µg/mL against *Pasteurella* and 0.5, 4, and >16 µg/mL against *Mannheimia* for CA, CT, and MA, respectively. CA, CT, and MA exhibited higher MIC_50_ and MIC_90_ values against *Pasteurella* isolates with a known AMR phenotype against commonly used therapeutic antimicrobial agents than against isolates with unknown AMR profiles. This study demonstrates the importance of whole-cell antibacterial screening of natural products to identify promising scaffolds with broad- or narrow-spectrum antimicrobial activity against important Gram-negative veterinary pathogens with zoonotic potential.

## 1. Introduction

Historically, natural products have played important roles as drugs in many therapeutic areas, including infectious diseases [1]. Following the discovery of antibiotics almost a century ago, several classes of antimicrobial agents of natural origin, such as β-lactams, tetracyclines, or aminoglycosides, were introduced as therapeutic agents. Antibiotic use, since then, has had an enormous impact on the treatment of infectious diseases in both human and veterinary medicine, but also on the ability to perform surgical procedures and immunosuppressing chemotherapy.

However, the ensuing frequent use of antimicrobial agents, including their misuse and overuse, has led to the concomitant emergence and dissemination of antimicrobial resistance (AMR) in bacteria [2,3,4,5,6], with the consequence that AMR is now considered one of the greatest threats to both human and animal health. In line with this, the Animal Health Law (Regulation (EU) 2016/429) states that “microorganisms that have developed resistance to antimicrobials should be treated as if they were transmissible diseases”. For 2019, predictive statistical models estimated a worldwide 4.95 million human deaths associated with bacterial AMR, thereof 1.27 million directly attributable to bacterial AMR [7]. Since antimicrobial administration to food animals and humans is a major driver of AMR and is projected to even increase in the future, bacterial AMR is anticipated to increase as well [8,9,10,11,12]. Thus, a continued presence of resistance determinants in commensal and pathogenic bacteria is the likely consequence of this uninterrupted selective pressure. Worldwide efforts have been and are being undertaken to address this threat [13,14]. In addition to antimicrobial stewardship programs and alternative novel and innovative treatment options [15], new antimicrobial agents will still be needed to combat AMR. Here, the role of natural products has declined in the current drug discovery process due to technical, economic, and intellectual property challenges [16], resulting in an antimicrobial agent pipeline that remains insufficient against priority pathogens [17,18]. The spectrum of chemical scaffolds used to develop novel antibacterial agents, thus, remains limited, so the discovery of new antimicrobial compound classes with novel modes of action is urgently needed [19].

A resurgence in natural product screening has been stimulated by recent progress in many different fields of research. These include not only bioinformatic analysis, genomics, and genome mining, as well as the exploitation of exotic ecological niches, but also involve improved understanding of AMR [20] and the development of new techniques to explore the chemistry of antimicrobial compounds [21]. Frequently identified with a specific research question in mind, most structures of interest are not routinely screened for activity against a broad panel of bacterial pathogens due to limited resources, especially in academia. Potential narrow-spectrum antimicrobials will, for example, almost certainly remain undiscovered this way. Examination of existing compound libraries is therefore still a promising strategy to identify new candidates for further development of novel antimicrobial drugs.

For the current study, we, therefore, selected eight substances out of six distinct chemotype classes from the natural product collection of the Leibniz-HKI based on, e.g., biotechnological accessibility, preliminary cell line toxicity, and primary bioactivity data. Particular emphasis was put on compounds with known anti-Gram-negative activity. All products selected were discovered in various ecological contexts, such as closthioamide, a unique polythioamide representing the first antibiotic found in an obligate anaerobic bacterium, or cervimycins from *Streptomyces* isolated from prehistoric cave wall paintings, which were made using bat dung [22,23].

The natural products selected were tested for antimicrobial activity against a diverse panel of bacterial species with importance as animal and/or zoonotic pathogens. For this purpose, we chose bacterial species that represent not only Gram-positive, Gram-negative, cell-wall-free, spore-forming, and acid-fast species, but also organisms with difficult-to-treat slow-growing or intracellular lifestyles. Additional criteria for choosing the bacterial species were (i) representing a relevant pathogen in human and/or veterinary medicine, (ii) the presence of frequently occurring AMR, an increasing problem for disease control [24], and (iii) the existence of established livestock animal models, which offers the possibility to evaluate the *in vivo* application of qualified substances at a later developmental stage (summarized in Table 1).

## 2. Results

The natural products were selected from the Leibniz-HKI natural compound collection, based on accessibility, data on preliminary activity, cytotoxicity, and individual properties. In total, eight natural products from six different compound classes exhibited promising characteristics. These products were screened following a two-stage process: First, in a pre-screening step, seven bacterial species were used to determine MIC (minimal inhibitory concentration) values. Based on the resulting activity profiles, we identified the most promising combinations of target species and test substances for secondary screening. Then, we determined the distribution of MIC values with a larger set of field isolates harboring varying resistance determinants.

**Figure 1:**
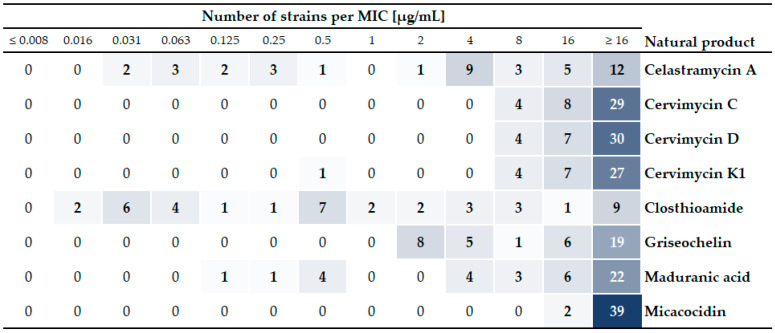
Heat map of the pre-screening results. Shown are the absolute numbers of isolates at their individual minimal inhibitory concentrations (MICs) for all eight substances. The heat map shows in bold the number of isolates combined from all bacterial species with a specific MIC value for each compound. Except for *C. suis* (*n* = 2 biological replicates), all isolates were tested once. The color intensity increases with the absolute number of strains identified to have a specific MIC value for a natural product.

**Figure 2:**
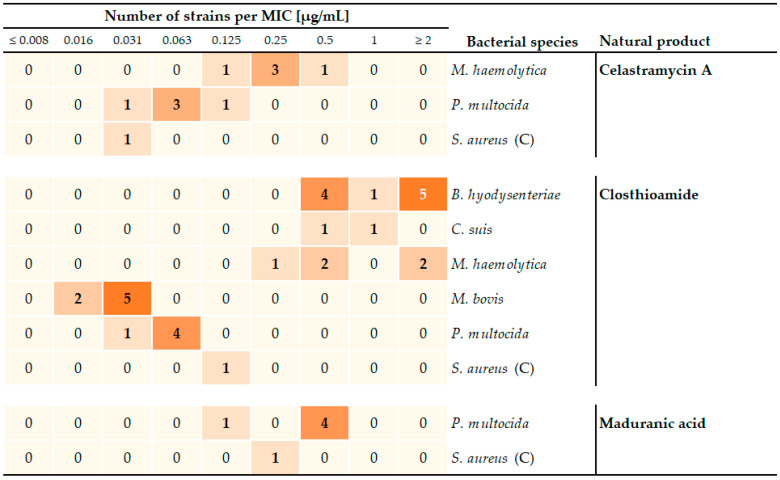
Heat map of the pre-screening results showing the most highly active natural products with MIC values ≤2 µg/mL that were evaluated for activity against isolates of Gram-positive, as well as Gram-negative or cell-wall-free bacterial species. The heat map shows in bold the number of isolates (from an individual bacterial species with a specific MIC value for each compound. Except for *C. suis* (*n* = 2 biological replicates), all isolates were tested once. The color intensity increases with the absolute number of strains identified to have a specific MIC value for a natural product.

The pre-screen results showed that the natural products inhibited growth in 5–78% of all isolates tested, with celastramycin A, closthioamide, and maduranic acid showing activity against several isolates at concentrations below 2 µg/mL (Figure 1). The remaining test compounds only inhibited growth in this concentration range in one control strain (cervimycin K1 in *S. aureus*) or, if at all, showed antibacterial activity at concentrations ≥2 µg/mL (Supplemental Table S1). In detail, micacocidin inhibited growth in two out of seven isolates of *M. bovis* at 16 µg/mL. Griseochelin and the cervimycin derivatives showed activity against two species, *C. difficile* and *M. bovis*, with MIC values of at least 4 µg/mL. Griseochelin was additionally active against *B. hyodysenteriae* at ≥2 µg/mL, while the cervimycin derivatives were additionally only active against one out of six isolates of the *M. avium* subspecies (8 µg/mL). In order to meet favorable pharmacokinetic/pharmacodynamic (PK/PD) relationships, low MIC values are preferred for further optimization in drug development. Closthioamide showed the broadest overall activity inhibiting the growth of isolates from six out of seven bacterial test species at relevant concentrations <2 µg/mL, followed by celastramycin A (three out of seven species), and maduranic acid (two out of seven species) (Figure 2). These compounds predominantly inhibited the growth of *P. multocida* field isolates, as well as the *S. aureus* control strain. In addition, celastramycin A, as well as closthioamide, inhibited *M. haemolytica* growth at concentrations below 1 µg/mL. Therefore, we selected the compounds celastramycin A, closthioamide, and maduranic acid for further screening.

**Figure 3:**
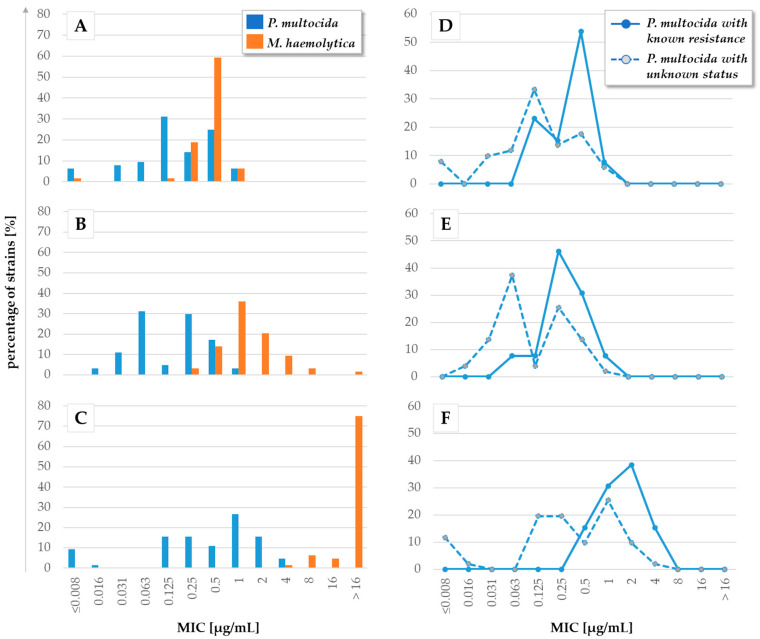
MIC values of the highly active natural products (**A**,**D**) celastramycin A, (**B**,**E**) closthioamide, and (**C**,**F**) maduranic acid against (**A**–**C**) field isolates of *P. multocida* (*n* = 64) and *M. haemolytica* (*n* = 56), as well as against (**D**–**F**) *P. multocida* isolates with (*n* = 13) known resistances against commonly used chemotherapeutics (see Supplemental Table S2) and *P. multocida* isolates that were previously not tested against commonly used chemotherapeutics (“unknown status”, *n* = 51). Results of broth microdilution assays.

*M. haemolytica* and *P. multocida* play major roles as causative agents of severe infections in farm animals that are frequently subject to antimicrobial treatment [46,47,48,49]. Compounds specifically targeting both pathogens are of particular interest. Therefore, field isolates of *P. multocida* (*n* = 64) and *M. haemolytica* (*n* = 56) collected from various cattle and pig sources (see Supplemental Table S2) were tested to determine MIC values for celastramycin A, closthioamide, and maduranic acid. As already indicated by the pre-screening results (Figure 2), growth inhibition generally required higher concentrations of the natural products in *M. haemolytica* than in *P. multocida* (Figure 3A–C). When applying a cut-off for MIC values higher than 1 µg/mL, celastramycin A showed growth-inhibition rates of 100% for *M. haemolytica*, as well as for *P. multocida*. The MIC_50_ and MIC_90_ values of celastramycin A determined against *M. haemolytica* were both 0.5 µg/mL, while the corresponding values against *P. multocida* were 0.125 and 0.5 µg/mL, respectively. The percentage of isolates showing inhibition determined for closthioamide in general (i.e., no cut-off applied) were 60.7% and 100% for *M. haemolytica* and *P. multocida*, respectively. In line with this observation, closthioamide showed higher MIC_50_ and MIC_90_ values against *M. haemolytica* with 1 and 4 µg/mL, respectively, while the equivalent values against *P. multocida* were the same as for celastramycin A with 0.125 and 0.5 µg/mL, respectively. In line with the pre-screening results, maduranic acid showed no activity against *M. haemolytica*, yet inhibited growth at concentrations below 2 µg/mL in 79.7% of the *P. multocida* isolates tested. The resulting MIC_50_ and MIC_90_ values were both higher than 16 µg/mL against *M. haemolytica*, and 0.5 and 2 µg/mL against *P. multocida*, respectively. The distribution of the MIC values of celastramycin A, closthioamide, and maduranic acid against *P. multocida* displayed a bimodal curve. When respective isolates of *P. multocida* are grouped by their resistance status (known or unknown), those isolates with known antibiotic resistance displayed, in most cases, higher MICs for celastramycin A, closthioamide and maduranic acid than the total average (Figure 3D–F).

## 3. Discussion

The emergence, evolution, and spread of AMR threaten global health systems, so multifaceted strategies, such as the 2015 WHO Global Action Plan on AMR, have become key elements in the effort to successfully combat AMR [50]. These include the screening of compounds to detect novel antimicrobial agents.

Of the eight compounds tested in the pre-screen, only celastramycin A, closthioamide, and maduranic acid showed promising antimicrobial activities at concentrations ≤2 µg/mL, with closthioamide inhibiting growth in most species (five out of seven). Closthioamide is, thus, the sole compound identified in this study with characteristics pointing towards a broad-spectrum antibiotic that is active against both Gram-negative and Gram-positive bacteria. In a previous study, closthioamide demonstrated activity against a broad spectrum of Gram-positive bacteria via the inhibition of DNA gyrase [51]. Celastramycin A and maduranic acid were active against fewer species at concentrations below 2 µg/mL. Two species, i.e., *M. haemolytica* and *P. multocida*, were inhibited by closthioamide and celastramycin A, while maduranic acid only inhibited growth in *P. multocida*. Both species frequently co-occur in respiratory diseases of farm animals [52,53]. While *P. multocida* has a broader host-range that also includes humans [54], *M. haemolytica* is predominantly pathogenic for ruminants [37]. Economic losses due to treatment costs and reduced weight gain in beef production [55,56], as well as decreased milk yield and other negative effects on dairy cow health [57,58,59], highlight the importance of treatment options for infections caused by these two pathogens; especially, since multidrug-resistant (MDR) isolates are increasingly isolated from cattle with bovine respiratory disease (BRD). In Germany, the rates of MDR isolates have increased more than six-fold in *P. multocida* within a five-year period (2015–2020) [60]. That study reported MDR rates of 13.9% and 5.1%, while rates of *pan*-susceptibility were only 15.1% and 12.1% in isolates of *P. multocida* and *M. haemolytica*, respectively [60]. Another recent study identified a novel high-level macrolide resistance determinant in *M. haemolytica*, further limiting treatment options for BRD [61]. New antimicrobial agents that target *P. multocida* and *M. haemolytica* would consequently be highly advantageous. Therefore, we enlarged the panel of bacterial isolates from these species by testing field isolates that originated from cattle and pigs (*P. multocida*) or only from cattle (*M. haemolytica*). Comparing the distribution of MIC values for the three compounds tested, isolates of *M. haemolytica* overall showed higher MIC values than those of *P. multocida*. As both genera are rather closely related, we have, so far, no physiological or mechanistic explanation for this observation. As an example, *M. haemolytica* isolates had higher MIC values than *P. multocida* isolates with respect to closthioamide and maduranic acid. The latter did not inhibit growth in *M. haemolytica*, yet reached a MIC_90_ of 2 µg/mL for *P. multocida*. As coinfections of *M. haemolytica* and *P. multocida* are frequently present in BRD, effective treatment requires agents targeting both species. Therefore, maduranic acid could be interesting for further investigation as a narrower-spectrum treatment option for infections with *P. multocida* other than BRD, especially due to its reported activity against Gram-positive bacteria [62]. Closthioamide showed activity against both species; however, the MIC_90_ of 4 µg/mL against *M. haemolytica* leaves only a narrow therapeutic window. The broad spectrum of bacterial species inhibited in growth by this compound (six out of seven) yet motivates further tests also against AMR-isolates of other relevant pathogens. Predestined for further testing would be, e.g., *Histophilus somni* and *M. bovis*, as in the context of BRD, both are the major bacterial agents of the syndrome besides *P. multocida* and *M. haemolytica* [52]. Further testing of closthioamide should therefore include field isolates of *M. bovis*, as closthioamide also displayed low MIC values against this species in the pre-screening, as well as isolates of *H. somni*, which was not part of this investigation, to cover the four most important bacterial pathogens from the BRD complex. Celastramycin A achieved the best results with MIC_90_ values for both panels of *P. multocida* and *M. haemolytica* field isolates. Moreover, celastramycin A has a modulatory effect on the immune system [63], like several other antibiotic classes (e.g., macrolides) [64,65,66], making this substance a promising candidate for further research.

While the MIC values for all three substances formed a single defined peak in all *M. haemolytica* isolates tested, isolates of *P. multocida*, in contrast, showed a bimodal MIC distribution. This observation partly reflects the classification of the isolates into two groups—with or without known AMR phenotype—and is most pronounced for maduranic acid. There, isolates with known resistance exclusively belong to the peak of MIC values ≥0.5 µg/mL. On the other hand, isolates with unknown status still form a bimodal distribution and, hence might consist of at least two subpopulations of phenotypes tolerating different concentrations of maduranic acid. The same observation is true for closthioamide, except for a few isolates with known resistance being susceptible at ≤0.125 µg/mL, and for celastramycin A, only less pronounced. A plausible explanation for this trend towards higher MIC values for the compounds tested against isolates with known resistance could be cross-resistance (i.e., possibly due to efflux), mediating higher tolerance towards the substances tested. This was previously reported for MDR isolates of *S. aureus*, which were able to tolerate higher doses of seaweed extract with antimicrobial activity than isolates with fewer resistance determinants [67]. Isolates with unknown resistance status, thus, should consist of (i) so-called wild-type (WT) isolates carrying no resistance determinants and hence tolerating only lower concentrations of test substances, and (ii) non-WT isolates that have acquired resistance determinants, hence tolerating higher concentrations [68]. A deeper analysis of the underlying mechanisms mediating resistance against these natural products could be helpful in identifying AMR determinants relevant for cross-resistance. Further investigation is required to determine the resistance profile of those isolates, which would allow us to clarify whether or not this explanation holds true.

## 4. Materials and Methods

### 4.1. Bacterial Isolates

A total of 41 bacterial reference (type and/or control) strains and field isolates from eight different families representing six phyla (Table 2) were pre-screened to determine bacterial susceptibility towards eight different natural products (Table 3). The respective isolates and their AMR profiles are summarized in Supplemental Table S1. Two strains (*E. coli* ATCC^®^ 25922 and *S. aureus* ATCC^®^ 29213), which were being used as controls, were single representatives of their species. Therefore, they were excluded from evaluating the activity testing of the included natural products.

Based on the results of the pre-screening, a second round of testing was performed in which an additional 51 *Mannheimia haemolytica* and 59 *Pasteurella multocida* field isolates were tested. These species included isolates with known resistance phenotypes against commonly used chemotherapeutic substances (*n* = 23 and *n* = 12, respectively), as well as isolates without information on their resistance profile (“unknown status”) (Supplemental Table S2). The isolates originated from farm animals (cattle and pigs) with no documented antibiotic treatment at least four weeks before sampling, and for some with a known clinical history. After revitalization from the cryo-conservation culture, all isolates, except the mycoplasma and chlamydia isolates, were at least twice freshly re-streaked on the respective agar prior to MIC testing.

### 4.2. Natural Products

The natural products to be tested were produced by Leibniz-HKI following published protocols (Table 3) and available as pure solid powders. Identity and purity were analyzed by LC–HRMS and HPLC, respectively, prior to dispatch. Stock solutions (10 mg/mL) of the substances were prepared in dimethyl sulfoxide (DMSO; Sigma-Aldrich, Germany). The respective working solutions (320 µg/mL) of cervimycin C, cervimycin D, cervimycin K1, closthioamide, griseochelin, micacocidin, and maduranic acid were prepared by diluting the respective stock solution 1:31.25 [*v*/*v*] in sterile, deionized H_2_O (diH_2_O). For celastramycin A, a first 1:10 [*v*/*v*] dilution step in diH_2_O/DMSO (1:1) was necessary, before it was further diluted 1:3.125 [*v*/*v*] in diH_2_O. Pre-testing of DMSO in the maximum working concentration used showed no inhibitory effects on the isolates themselves. Prior to their use, all working solutions were 1:10 [*v*/*v*] diluted in the respective test medium. Due to limited amounts of the natural products and the search for highly potent active ingredients, the natural products were tested for their antimicrobial activity up to a concentration of 16 µg/mL.

### 4.3. Determination of Minimal Inhibitory Concentrations (MICs) of the Natural Products

For most bacterial species to be tested, the MIC values were determined using broth microdilution methodology (Table 2). To this end, CLSI protocols (broth microdilution dilution and agar dilution assays) were followed as closely as possible [80] with only minor modifications as specified below. Each test protocol included sterility (medium without bacteria and natural products) and growth controls (medium with bacteria but without natural products).

The MIC values for the quality control strains *Escherichia* (*E.*) *coli* strain ATCC 25922 and *Staphylococcus* (*S.*) *aureus* ATCC 29213 were determined following the CLSI protocol Vet01-A4 [81]. In brief, the control strains were grown on 5% sheep blood agar plates (35 °C, 16–18 h) and 3–5 single colonies were suspended in 5 mL sterile 0.9% NaCl solution, corresponding to a 0.5 McFarland standard; thereof, 50 µL were diluted in 11 mL Müller-Hinton broth (MHB; Oxoid, Germany) and served as inoculum containing approx. 5 × 10^5^ cfu/mL. The cfu of the inoculum was controlled by diluting each inoculum 1:1000 in sterile 0.9% NaCl solution and plating 100 µL of the diluted inoculum on a sheep blood agar plate. After incubation (37 °C, 18–24 h), colonies were counted and the cfu in the inoculum was calculated. The MIC testing was performed in 96-U-well PS-microtiter plates (Greiner Bio-One, Germany) with 100 µL of the natural products freshly diluted in MHB and 100 µL of the inoculum. After incubation (35 °C, 16–20 h), the wells were checked visually for bacterial growth and the MIC determined as the lowest concentration of the natural product that completely inhibited bacterial growth.

MIC testing of *Pasteurellaceae* was carried out as described above with the following modifications: the isolates were freshly grown on 5% sheep blood agar plates (37 °C, 20–24 h) and 5 single colonies were suspended in 5 mL sterile 0.9% NaCl solution. Thereof, 400 µL were diluted in 11 mL Cation-Adjusted MHB (CAMHB; Oxoid, Germany) and used as inoculum with approx. 5 × 10^5^ cfu/mL. The cfu counts of the inoculum were controlled as described above. MIC testing was performed as described above, but with CAMHB instead of MHB and incubation of the microtiter plates at 37 °C for 20–24 h.

*Mycobacterium avium* subsp. *avium* (MAA) and subsp. *hominissuis* (MAH) were tested following the CLSI protocol M24-A02 [82]. The isolates were grown on Löwenstein–Jensen agar slants (with glycerol, without pyruvate, Artelt-Enclit, Borna, Germany). Thereof, colony material was collected and resuspended in 5 mL sterile 0.9% NaCl solution. To resolve agglomerated bacteria, 3–5 glass beads were added followed by vortexing the suspension for 10–20 s. Then, 100 µL were transferred to 11 mL CAMHB with 5% oleic acid–albumin–dextrose–catalase solution (OADC, Sigma-Aldrich, Germany). Inoculation of the microtiter plates and cfu control of the inoculum on Middlebrook 7H11 agar plates with OADC (Sigma-Aldrich, Germany) were performed as described above. The incubation period of the microtiter and agar plates was extended to 7–10 d (35 °C).

The MIC testing of *Brachyspira* (*B.*) *hyodysenteriae* isolates was adapted to the in-house protocol published by Herbst et al., 2014 [27]. *B. hyodysenteriae* isolates were grown on tryptic soy agar plates with 5% sheep blood (TSB) under anaerobic conditions (37 °C, 3–5 d). To prepare the inoculum, 5 loops with bacteria were resuspended in 14 mL brain heart infusion broth supplemented with 20% fetal calf serum (BHIF; Becton Dickinson, Heidelberg, Germany; Biochrom, Berlin, Germany) to reach 10^5^ cfu/mL. The inoculum was controlled by plating log10 dilutions on TSB and checking for growth of *B. hyodysenteriae* indicated by strong hemolysis. The natural products were diluted in BHIF and 100 µL per dilution step were dispensed into 96-U-well PS-microtiter plates. After adding 100 µL of the inoculum, the microtiter plates were incubated (anaerobic, 37 °C, 5 d, shaking [130 rpm]). Using a dark light table with lateral light fall, the MIC was determined as the macroscopically visible lowest concentration that completely inhibited bacterial growth.

For titration and MIC analyses, *Mycoplasma* (*M.*) *bovis* isolates were grown in commercially available ML10 broth (Mycoplasma Experience, Redhill, UK) without antimicrobial agents and supplemented with sodium pyruvate (0.5%) and phenol red (0.005%) as described by Hannan, 2000 [69]. Test culture batches were prepared for each isolate through 48 h growth in ML10, which were then aliquoted and stored frozen at −80 °C. The number of color-changing units (CCUs) was determined via broth microdilution [83]. The natural products were diluted in ML10 broth and 100 µL applied to each well on microtiter plates. The inoculum of 10^4^ CCU/mL was prepared from titrated *M. bovis* culture batches and freshly cultured for 2 h at 37 °C before 100 µL were applied to each well to give a final concentration of 5 × 10^5^ CCU/mL. Individual isolates were tested at least twice. Plates were sealed with a sterile plastic cover, incubated at 37 °C, and analyzed after 40–48 h, as soon as a color shift became evident in the wells lacking any natural products that served as growth controls. MIC values were determined as the lowest concentration of a natural product that completely suppressed *M. bovis* growth. When individual MIC values for an isolate differed by a maximum factor of two, the higher concentration was recorded, otherwise, testing was repeated.

Two *Chlamydia* (*C*.) *suis* isolates with known tetracycline resistance (see Supplemental Table S1) were used to determine the inhibitory effect of the natural products on intracellular bacteria. The natural products were pre-diluted in Ultra-MDCK medium (Lonza, Basel, Switzerland) in a final volume of 100 µL per well and applied to microtiter plates. For the inoculum, 100 µL of a buffalo green monkey (BGM) cell suspension containing 4 × 10^4^ cells in Ultra-MDCK and chlamydiae adjusted to a multiplicity of infection (MOI) of 0.05 were added. Plates were centrifuged at 2000× *g* and 37 °C for 1 h and incubated at 37 °C, 5% CO_2_ for 30–32 h. Then, the medium was removed and the cells were fixed with 200 µL methanol per well overnight and stained using the IMAGEN Chlamydia kit (Oxoid, Wesel, Germany) according to the manufacturer’s instructions. Fluorescent inclusion forming units (IFUs) were counted under a fluorescence microscope and the MIC was defined as the lowest concentration of the natural product preventing the detection of more than 90% of the chlamydial inclusions compared with the drug-free control. All tests were run in duplicate.

The MIC profiles for *Clostridioides* (*C.*) *difficile* were determined following the Wadsworth method in document M11-A8 [84] as an agar dilution assay. Briefly, enriched Brucella agar (Carl Roth, Germany) plates, additionally containing vitamin K [1 µg/mL], haemin [5 µg/mL], and defibrinated sheep blood [5%] (Oxoid, Germany), were prepared and supplemented with the natural products in a log2 dilution series ranging from 16 µg/mL to 0.0675 µg/mL. Assays were performed in Petri dishes or 6-well cell culture plates. The *C. difficile* isolates were cultivated on enriched Brucella agar and incubated under anaerobic conditions (37 °C, 48 h). To prepare the suspension used to inoculate the plates, 3 to 7 single *C. difficile* colonies were suspended in 5 mL Brucella broth (Carl Roth, Karlsruhe, Germany) and 1.5 µL of the inoculum (corresponding to 5 × 10^5^ cfu/spot) spotted on enriched and supplemented Brucella agar plates, as well as on enriched Brucella agar without supplemented natural products. After the drops had dried, the plates were inverted and incubated (anaerobic conditions, 37 °C, 42 to 48 h). The MIC was defined as the dilution step at which a marked reduction in colony growth is macroscopically visible compared to the control plate without supplemented natural products. The cfu of the inoculum was controlled each test day by plating 100 µL of a 1:1000 dilution of the inoculum in sterile 0.9% NaCl solution on an enriched Brucella agar plate and colony counting after incubation (anaerobic conditions, 37 °C, 42 to 48 h).

## 5. Conclusions

The study carried out here shows that natural products still yield interesting and worthwhile candidates for the development of antibiotically active therapeutics. This study also shows that it is important that the screening of natural products covers a spectrum of target pathogens/species as broad and heterogeneous as possible (e.g., Gram-positive, Gram-negative, cell-wall-free, spore-forming, and acid-fast species and/or intracellular growth), including field and clinical isolates, in order to be able to fully evaluate the true potential of the compounds.

## Figures and Tables

**Table 1:** Background information on the bacterial species included in the current study concerning the diseases they cause in livestock, their most relevant AMR, and available infection models.

Bacterial Species	Infectious Lifestyle	Disease in Animals (Most Relevant Hosts)	Reference	Antimicrobial Resistance (AMR)		Animal Model (Host, Organ System)	
				Antibiotic Class Affected	Reference	Species	Reference
*Brachyspira* *hyodysenteriae*	Extracellular	Dysentery (Swine)	Hampson et al., 2019 [25]	Macrolides, Pleuromutilins	Hampson et al., 2019 [26] Herbst et al., 2014 [27]	Swine, Intestinal tract	La et al., 2019 [28]
*Chlamydia suis*	Intracellular	Respiratory disease, Diarrhea, Conjunctivitis (Swine)	Rogers et al., 1999 [29] Reinhold et al., 2008 [30] Guscetti et al., 2009 [31]	Tetracyclines	Bommana et al., 2019 [32]	Swine, Respiratory tract	Reinhold et al., 2011 [33]
*Clostridioides* *difficile*	Extracellular	Diarrhea (Piglets, Horses)	Weese, 2020 [34]	Fluoroquinolones, Macrolides, Ansamycins	Dureja et al., 2022 [35]	Swine, Intestinal tract	Steele et al., 2010 [36]
*Mannheimia* *haemolytica*	Extracellular and facultative intracellular	Respiratory disease (Cattle, Goats, Sheep)	Michael et al., 2018 [37]	β-Lactams, Macrolides, Tetracyclines	Michael et al., 2018 [37]	Cattle, Respiratory tract	Schroedl et al., 2001 [38]
*Mycobacterium avium* ssp.	Intracellular	Respiratory disease, Johne’s disease (Cattle)	Fecteau, 2018 [39]	Macrolides, Ansamycins	Saxena et al., 2021 [40]	Goat, Intestinal tract	Köhler et al., 2015 [41]
*Mycoplasma* *bovis*	Extracellular	Respiratory disease, Mastitis, Arthritis (Cattle)	Dudek et al., 2020 [42]	Macrolides, Phenicols, Tetracyclines	EFSA Panel on Animal Health and Welfare et al., 2021 [24]	Cattle, Respiratory tract	Dudek et al., 2019 [43]
						Cattle, Mammary gland	Byrne et al., 2005 [44]
*Pasteurella* *multocida*	Extracellular and facultative intracellular	Respiratory disease (Cattle, Sheep, Swine), Mastitis (Sheep), Septicaemia (Cattle)	Michael et al., 2018 [37]	β-Lactams, Macrolides, Tetracyclines	Michael et al., 2018 [37]	Cattle, Respiratory tract	Reinhold et al., 2002 [45]

**Table 2:** Bacterial isolates used for pre-screening of the natural products.

Bacterial Species	Family	Phylum	Cellular Properties	Test System	Reference	No. of Type (T) or Control (C) Strains ^1^	No. of Field Isolates
*Brachyspira hyodysenteriae*	*Brachyspiraceae*	Spirochaetota	Gram negative	BMD	Herbst et al., 2014 [27]	-	10
*Chlamydia suis*	*Chlamydiaceae*	Chlamydiota	Gram negative	BMD	In-house method (cell culture with BGM cells, see Section 4.3)	1 (T)	1
*Clostridioides difficile*	*Peptostreptococcaceae*	Bacillota	Gram positive, spore forming	AD	CLSI M11-A08	-	4
*Escherichia coli*	*Enterobacteriaceae*	Pseudomonadota	Gram negative	BMD	CLSI Vet01 A4	1 (C)	-
*Mannheimia haemolytica*	*Pasteurellaceae*	Pseudomonadota	Gram negative	BMD	CLSI Vet01 A4	-	5
*Mycobacterium avium* ssp.	*Mycobacteriaceae*	Actinomycetota	Acid-fast bacteria	BMD	CLSI M24-A02	1 (T), 1 (C)	4
*Mycoplasma bovis*	*Mycoplasmataceae*	Mycoplasmatota	Cell-wall free	BMD	Hannan, 2000 [69]	1 (T)	6
*Pasteurella multocida*	*Pasteurellaceae*	Pseudomonadota	Gram negative	BMD	CLSI Vet01 A4	-	5
*Staphylococcus aureus*	*Staphylococcaceae*	Bacillota	Gram positive	BMD	CLSI Vet01 A4	1 (C)	-

Annotations: AD, agar dilution assay; BMD, broth microdilution assay; BGM, buffalo green monkey cells. ^1^ type strain as stated by the ATCC, control strains are recommended by the respective CLSI protocol.

**Table 3:** Summary of natural products and their known characteristics.

Natural Product	Origin	Structure	MW [g/mol]	Production	Mode of Action	Toxicity CC_50_ [mg/L] LD_50_ [mg/kg]	Reference
Celastramycin A	*Streptomyces* MaB-QuH-8	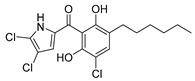	390.69	Synthetic	Not known	CC_50_ 16.9 ^a^	Kikuchi et al., 2009 [70]; Pullen et al., 2002 [71]
Closthioamide	*Ruminiclostridium cellulolyticum*	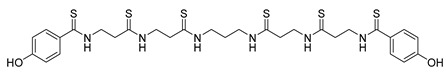	695.04	Synthetic	Inhibition of topoisomerase II	CC_50_ 5.2 ± 0.5 ^a^	Chiriac et al., 2015 [51]; Lincke et al., 2010 [72]
Cervimycin C	*Streptomyces tendae* HKI-179	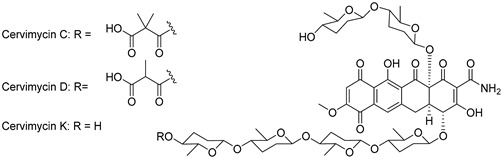	1228.29	Microbial	Not known	CC_50_ >50 ^a^	Herold et al., 2004 [23]; Herold et al., 2005 [22]
Cervimycin D	*Streptomyces tendae* HKI-179		1214.26	Microbial	Not known	CC_50_ >50 ^a^	Herold et al., 2004 [23]; Herold et al., 2005 [22]
Cervimycin K1	*Streptomyces tendae* HKI-179		1114.19	Microbial	Not known	CC_50_ 11.0 ^a^	Dietrich et al., 2022 [73]
Griseochelin	*Streptomyces griseus * HKI 0741	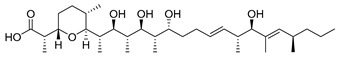	568.83	Microbial	Not known, but ionophore (Zn^2+^) activity	CC_50_ 0.6 ± 0.3 ^b^; 13.7 ± 15.4 ^c^	Gräfe et al., 1984 [74]; Walther et al., 2016 [75]
Micacocidin	*Ralstonia solanacearum* GMI 1000 and *Pseudomonas* sp. No. 57-250	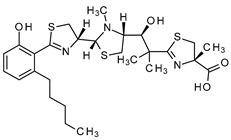	565.81	Microbial	Not known	CC_50_ >50 ^a^	Ino et al., 1998 [76]; Kage et al., 2013 [77]
Maduranic acid/madurahdroxylacton	*Nonomuria rubra* DSM12263	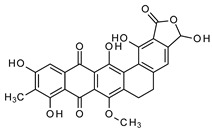	490.42	Microbial	Suggested FtsZ inhibitor	LD_50_ >250 ^d^	Fleck et al., 1978 [78]; Kim et al., 2017 [79]

Annotations: CC_50_, concentration of the respective natural product to reduce cell viability by 50%; FtsZ, filamenting temperature-sensitive mutant Z protein, a prokaryotic homolog of the eukaryotic protein tubulin, involved in cell division; LD_50_, lethal dose, concentration of the respective natural product to kill 50% of the tested animal population; MW, molecular weight. ^a^ in HeLa cells; ^b^ in A549 cells; ^c^ in MDCK cells; ^d^ in mice (intraperitoneal).

## Data Availability

The data presented in this study are included in the Supplemental Material and openly available in Zenodo at https://zenodo.org/records/10214242 (created on 4 December 2023).

## Supplementary Materials

The following supporting information can be downloaded at https://www.mdpi.com/article/10.3390/antibiotics13020135/s1, Table S1: Results of pre-screening the natural products against different bacterial species; Table S2: Results of testing additional *Mannheimia haemolytica* and *Pasteurella multocida* isolates. References [85,86,87,88] are cited in the supplemenatry materials.
