# Supplementary material for: In Vitro Antibacterial Activity of Microbial Natural Products against Bacterial Pathogens of Veterinary and Zoonotic Relevance
Source: Antibiotics (Basel). 2024 Jan 30;13(2):135. doi: 10.3390/antibiotics13020135 (PMC10886079; doi:10.3390/antibiotics13020135)
Supplement: Supplementary file 1 [file antibiotics-13-00135-s001.zip › antibiotics-2787557-supplementary.pdf]

Supplemental Material

In Vitro Antibacterial Activity of Microbial Natural Products against Bacterial Pathogens of Veterinary and Zoonotic Relevance

Stefanie A. Barth [1,*], Daniel Preussger [1], Jana Pietschmann [1], Andrea T. Feßler [2,3], Martin Heller [1], Werner Herbst [4], Christiane Schnee [1], Stefan Schwarz [2,3], Florian Kloss [5], Christian Berens [1] and Christian Menge [1]

1 Friedrich-Loeffler-Institut—Federal Research Institute for Animal Health (FLI), Institute of Molecular Pathogenesis, 07743 Jena, Germany
2 Institute of Microbiology and Epizootics, Freie Universität Berlin, 14163 Berlin, Germany
3 Veterinary Centre for Resistance Research (TZR), Freie Universität Berlin, 14163 Berlin, Germany
4 Institute of Hygiene and Infectious Diseases of Animals, Justus-Liebig-University, 35392 Giessen, Germany
5 Transfer Group Anti-Infectives, Leibniz Institute for Natural Product Research and Infection Biology, Leibniz-HKI, 07745 Jena, Germany
* Correspondence: stefanie.barth@fli.de

Content

Table S1. Results of pre-screening the natural products against different bacterial species.

Table S2. Results of testing additional *Mannheimia haemolytica* and *Pasteurella multocida* isolates, previously tested for resistance against common antibiotics, for growth or growth inhibition in the presence of natural products celastramycin A, maduranic acid, and closthioamide. All strains are isolated from animals without prior antibiotic treatment, at least for four weeks.

Table S1. Results of pre-screening the natural products against different bacterial species

Bacterial species	Isolate ID	Host species or reference[1]	Resistance profile[2]	Source[3]	Minimal inhibitory concentration (MIC) [μg/mL]							
					Celastra-mycin A	Cervi-mycin C	Cervi-mycin D	Cervi-mycin K1	Closthio-amide	Griseo-chelin	Madu-ranic acid	Micaco-cidin
Escherichia coli	ATCC 25922	control strain	unknown status	DSMZ	>16	>16	>16	>16	>16	>16	>16	>16
Staphylococcus aureus	ATCC 29213	control strain	unknown status	DSMZ	0.031	>16	>16	0.5	0.125	>16	0.25	>16
Clostridioides difficile	10S0042/RT660	cattle	unknown status	FLI-IBIZ	4	8	8	8	>16	4	>16	>16
	11S0095/RT620	cattle	unknown status	FLI-IBIZ	4	8	8	8	>16	4	>16	>16
	11S0044/RT078	pig	unknown status	FLI-IBIZ	4	8	8	8	>16	4	>16	>16
	12S0090/RT002	pig	unknown status	FLI-IBIZ	4	8	8	8	>16	4	>16	>16
Mycobacterium avium ssp. *avium*	ATCC 25291[T]	type strain	unknown status	DSMZ	8	>16	>16	>16	16	>16	16	>16
Mycobacterium avium ssp. *hominissuis*	ATCC 700898	control strain	unknown status	ATCC	4	>16	>16	>16	8	>16	16	>16
	00A0854	cattle	unknown status	FLI-IMP	4	>16	>16	>16	>16	>16	>16	>16
	00A0799	cattle	unknown status	FLI-IMP	4	16	16	16	8	>16	8	>16
	03A2893	cattle	unknown status	FLI-IMP	4	>16	>16	>16	>16	>16	>16	>16
	09MA1289	pig	unknown status	FLI-IMP	8	16	>16	>16	4	>16	4	>16
Mycoplasma bovis	PG45[T]	type strain	unknown status	FLI-IMP	16	16	16	16	0.031	8	16	16
	393B08	cattle	TYL, TIL, LIN, CLI, ERY, CHL, FLO, SPT, TET, ENR, MAR, TUL	FLI-IMP/VLA	4	16	16	16	0.016	16	16	>16
	216B09	cattle	TYL, TIL, LIN, CLI, ERY, CHL, FLO, SPT, TET, TUL	FLI-IMP/VLA	16	16	16	16	0.031	16	>16	16

Bacterial species	Isolate ID	Host species or reference[1]	Resistance profile[2]	Source[3]	Minimal inhibitory concentration (MIC) [µg/mL]							
					Celastra-mycin A	Cervi-mycin C	Cervi-mycin D	Cervi-mycin K1	Closthio-amide	Griseo-chelin	Madu-ranic acid	Micaco-cidin
	369B09	cattle	TYL, TIL, LIN, CLI, ERY, CHL, FLO, SPT, TET, ENR, MAR, TUL	FLI-IMP/VLA	16	16	>16	>16	0.031	16	>16	>16
	263B09	cattle	TIL, ERY, CHL, SPT, TET	FLI-IMP/VLA	16	16	16	16	0.016	16	16	>16
	410B09	cattle	TIL, ERY, CHL, FLO, TET	FLI-IMP/VLA	16	>16	>16	16	0.031	16	>16	>16
	268B07	cattle	TIL, ERY, CHL	FLI-IMP/VLA	8	16	16	16	0.03	16	>16	>16
Mannheimia haemolytica	P649	cattle	TET	FLI-ING	0.125	>16	>16	>16	0.25	>16	4	>16
	M3	cattle	TET	FLI-ING	0.5	>16	>16	>16	0.5	>16	8	>16
	667	cattle	AMP, TET	FLI-ING	0.25	>16	>16	>16	0.5	>16	>16	>16
	652	cattle	susceptible	FLI-ING	0.25	>16	>16	>16	>16	>16	16	>16
	650	cattle	susceptible	FLI-ING	0.25	>16	>16	>16	2	>16	8	>16
Pasteurella multocida	B624	pig	TET	FLI-ING	0.125	>16	>16	>16	0.063	>16	0.5	>16
	O6551	cattle	susceptible	FLI-ING	0.063	>16	>16	>16	0.031	>16	0.125	>16
	B368	not known	susceptible	FLI-ING	0.031	>16	>16	>16	0.063	>16	0.5	>16
	H301	not known	susceptible	FLI-ING	0.063	>16	>16	>16	0.063	>16	0.5	>16
	H287	not known	susceptible	FLI-ING	0.063	>16	>16	>16	0.063	>16	0.5	>16
Brachyspira hyodysenteriae	ATCC 49526	pig	TYL	JLU-IHIT	>16	>16	>16	>16	4	2	>16	>16
	B204	pig	TYL	JLU-IHIT	>16	>16	>16	>16	8	2	>16	>16
	G385	pig	TYL	JLU-IHIT	>16	>16	>16	>16	0.5	2	>16	>16

Bacterial species	Isolate ID	Host species or reference[1]	Resistance profile[2]	Source[3]	Minimal inhibitory concentration (MIC) [µg/mL]							
					Celastra-mycin A	Cervi-mycin C	Cervi-mycin D	Cervi-mycin K1	Closthio-amide	Griseo-chelin	Madu-ranic acid	Micaco-cidin
	G504	pig	TYL	JLU-IHIT	>16	>16	>16	>16	>16	>16	>16	>16
	G296	pig	susceptible	JLU-IHIT	>16	>16	>16	>16	4	2	>16	>16
	G347	pig	susceptible	JLU-IHIT	>16	>16	>16	>16	0.5	2	>16	>16
	G367	pig	TIA, VAL	JLU-IHIT	>16	>16	>16	>16	0.5	4	>16	>16
	G376	pig	TIA, VAL	JLU-IHIT	>16	>16	>16	>16	2	2	>16	>16
	G382	pig	susceptible	JLU-IHIT	>16	>16	>16	>16	1	2	>16	>16
	G387	pig	susceptible	JLU-IHIT	>16	>16	>16	>16	0.5	2	>16	>16
Chlamydia suis	$S45^T$	type strain	susceptible	FLI-IMP	>16	>16	16	n.t.	1	n.t.	4	>16
	DC127	pig	TET	FLI-IMP	2	>16	>16	n.t.	0.5	n.t.	4	>16

Annotations: 1) **Type strains** as stated by the ATCC strain collection, **control strains** are recommended by the respective CLSI protocol.
2) **AMP,** ampicillin (aminopenicillin β-lactam); **CHL,** chloramphenicol (amphenicol); **CLI,** clindamycin (lincosamide); **ENR,** enrofloxacin (quinolone); **ERY,** erythromycin (macrolide); **FLO,** florfenicol (amphenicol); **LIN,** lincomycin (lincosamide); **MAR,** marbofloxacin (quinolone); **SPT,** spectinomycin (aminocyclitol); **TET,** tetracycline; TIA, tiamulin (pleuromutilin); **TIL,** tilmicosin (macrolide); **TUL,** tulathromycin (macrolide); **TYL,** tylosin (macrolide); **VAL,** valnemulin (pleuromutilin).
3) **ATCC,** American type culture collection, U.S.A.; **DSMZ,** Deutsche Sammlung von Mikroorganismen und Zellkulturen, Germany; **FLI,** Friedrich-Loeffler-Institut - Federal Research Institute for Animal Health; **IBIZ,** Institute of Bacterial Infections and Zoonoses; **IMP,** Institute of Molecular Pathogenesis; **ING;** Institute of Farm Animal Genetics; **JLU-IHIT,** Institute of Hygiene and Infectious Diseases of Justus-Liebig-University Giessen; **VLA,** Veterinary Laboratories Agency, UK.
n.t., not tested.

Table S2. Results of testing additional *Mannheimia haemolytica* and *Pasteurella multocida* isolates, previously tested for resistance against common antibiotics, for growth or growth inhibition in the presence of natural products celastramycin A, maduranic acid, and closthioamide. All strains are isolated from animals without prior antibiotic treatment, at least for four weeks.

Isolate	Origin/ clinical status[1]	Resistance profile[2]	Minimal inhibitory concentration (MIC) [µg/mL]		
			Celastra-mycin A	Maduranic acid	Closthio-amide
Mannheimia haemolytica					
A5667/2	cattle/D	TET, STR, AMP, SXT, TMP	0.5	>16	0.5
A5747	cattle/D	TET, STR, AMP	0.5	>16	0.5
M166	cattle/D	TET, STR, AMP, CHL	0.5	>16	4
M395	cattle/D	TET, STR	0.5	>16	1
M55	cattle/D	TET, STR, AMP, CHL	0.25	>16	2
P658	cattle/D	TET, STR, AMP	0.5	>16	1
R130	cattle/D	TET, STR	0.5	>16	2
R140	cattle/D	TET, STR, AMP, CHL	0.5	>16	4
R141	cattle/D	TET, STR, AMP, CHL	0.25	>16	1
R144	cattle/D	TET, STR, AMP, CHL	0.5	>16	2
R241	cattle/D	TET, STR, AMP	0.25	>16	1
R47	cattle/D	TET, STR, AMP	0.25	>16	1
U-B143	cattle/D	TET, STR	0.5	>16	1
U-B144	cattle/D	TET, STR, AMP	0.5	>16	2
U-B352	cattle/D	TET, STR	≤0.008	8	0.25
U-B375	cattle/D	TET, STR, AMP	0.5	>16	1
U-B379	cattle/D	TET, STR, AMP, GEN	0.5	>16	2
U-B386	cattle/D	TET, STR, CHL, KAN	0.5	>16	0.5
U-B65	cattle/D	TET, STR, AMP	0.5	>16	1
U-B67	cattle/D	TET, STR, AMP	1	>16	1
2512	cattle/H	TET, STR, AMP, CHL, GEN, KAN, SUL, TMP	1	>16	8
3242	cattle/H	TET, STR, SUL	0.5	>16	1
3259	cattle/H	TET, STR, AMP, CHL	0.5	>16	1
5577	cattle/nk	unknown status	0.5	16	1
5578	cattle/nk	unknown status	0.25	>16	1
5838	cattle/nk	unknown status	0.25	>16	1
5848	cattle/nk	unknown status	0.5	>16	2
5940	cattle/nk	unknown status	0.25	>16	1
5941	cattle/nk	unknown status	0.5	>16	0.5
5952	cattle/nk	unknown status	0.5	>16	1
5953	cattle/nk	unknown status	0.5	>16	1
6040	cattle/nk	unknown status	0.5	>16	2
6086	cattle/nk	unknown status	0.5	16	2
6091	cattle/nk	unknown status	0.5	>16	1
6105	cattle/nk	unknown status	0.25	>16	0.5
6320	cattle/nk	unknown status	0.5	>16	1
6355	cattle/nk	unknown status	1	>16	4
6356	cattle/nk	unknown status	0.25	8	1
6401	cattle/nk	unknown status	0.5	>16	0.5
6467	cattle/nk	unknown status	0.5	>16	1
6475	cattle/nk	unknown status	0.5	>16	4
6884	cattle/nk	unknown status	0.5	>16	4
6888	cattle/nk	unknown status	1	>16	2

Isolate	Origin/ clinical status[1]	Resistance profile[2]	Minimal inhibitory concentration (MIC) [µg/mL]		
			Celastra-mycin A	Maduranic acid	Closthio-amide
454	cattle/nk	unknown status	0.5	>16	2
637	cattle/nk	unknown status	0.5	>16	4
686	cattle/nk	unknown status	0.5	>16	2
688	cattle/nk	unknown status	0.5	>16	0.5
708	cattle/nk	unknown status	0.5	>16	1
731	cattle/nk	unknown status	0.5	>16	8
781	cattle/nk	unknown status	0.5	>16	2
786	cattle/nk	unknown status	0.5	>16	1
Pasteurella multocida					
B444	pig/nk	TET, STR	0.125	1	0.25
B61	pig/nk	TET, STR	0.25	2	0.25
B71a	pig/nk	TET, STR, KAN, GEN	0.25	2	0.25
B87/99	pig/nk	TET, STR, GEN, SXT, TMP	1	2	0.5
H151	cattle/nk	TET, STR, KAN, AMP, GEN, SXT, TMP	0.5	4	0.25
H3152	pig/D	TET, STR, KAN	0.125	1	0.5
H468	pig/nk	TET, STR, SXT, TMP	0.5	2	0.125
U-B214	cattle/D	TET, STR, AMP	0.5	1	0.5
U-B447	cattle/D	TET, STR, KAN, AMP, SXT, TMP	0.5	1	1
U-P207	pig/D	TET, STR	0.5	2	0.25
623	pig/nk	unknown status	0.125	2	0.5
640	pig/nk	unknown status	0.5	4	0.25
687	pig/nk	unknown status	0.5	2	0.25
694	pig/nk	unknown status	0.25	1	0.25
721	pig/nk	unknown status	0.25	1	0.5
1142	pig/nk	unknown status	0.25	1	0.25
1148	pig/nk	unknown status	1	1	0.25
1179	pig/nk	unknown status	0.25	1	0.063
1271	pig/nk	unknown status	0.125	1	0.25
1403	pig/nk	unknown status	0.25	0.5	0.25
1450	pig/nk	unknown status	0.5	1	0.25
1057	cattle/nk	unknown status	0.125	0.25	0.063
1175	cattle/nk	unknown status	0.5	2	0.5
1198	cattle/nk	unknown status	≤0.008	≤0.008	0.03
1275	cattle/nk	unknown status	0.5	1	0.25
1589	cattle/nk	unknown status	≤0.008	≤0.008	0.031
5465	cattle/nk	unknown status	0.125	0.125	0.031
5466	cattle/nk	unknown status	≤0.008	0.25	0.063
5492	cattle/nk	unknown status	0.125	0.25	0.031
5521	cattle/nk	unknown status	0.125	0.015	0.015
5575	cattle/nk	unknown status	0.063	≤0.008	0.063
5579	cattle/nk	unknown status	0.031	≤0.008	0.063
5580	cattle/nk	unknown status	0.031	≤0.008	0.063
5868	cattle/nk	unknown status	0.031	0.125	0.031
5925	cattle/nk	unknown status	0.25	0.125	0.063
5928	cattle/nk	unknown status	0.125	0.25	0.063
5933	cattle/nk	unknown status	0.063	0.125	0.125
5934	cattle/nk	unknown status	0.25	0.25	0.25
5935	cattle/nk	unknown status	1	1	1
5939	cattle/nk	unknown status	0.125	0.25	0.063
6002	cattle/nk	unknown status	0.125	0.25	0.125

Isolate	Origin/ clinical status[1]	Resistance profile[2]	Minimal inhibitory concentration (MIC) [µg/mL]		
			Celastra-mycin A	Maduranic acid	Closthio-amide
6034	cattle/nk	unknown status	0.063	0.125	0.063
6118	cattle/nk	unknown status	0.125	0.125	0.063
6193	cattle/nk	unknown status	1	2	0.5
6255	cattle/nk	unknown status	0.125	0.25	0.063
6304	cattle/nk	unknown status	0.125	0.125	0.063
6592	cattle/nk	unknown status	0.125	0.25	0.031
2481	cattle/nk	TET, STR, KAN, GEN, SUL	0.5	4	0.5
1007	cattle/nk	TET, STR, KAN, GEN, SPT, SUL	0.5	0.5	0.25
7	cattle/nk	unknown status	0.125	0.25	0.25
63	cattle/nk	unknown status	0.125	1	0.5
128	cattle/nk	unknown status	0.031	0.125	0.063
256	cattle/nk	unknown status	0.5	1	0.5
257	cattle/nk	unknown status	0.5	1	0.25
456	cattle/nk	unknown status	0.5	1	0.25
636	cattle/nk	unknown status	0.125	0.5	0.063
681	cattle/nk	unknown status	0.5	2	0.5
683	cattle/nk	unknown status	≤0.008	≤0.008	0.015
684	cattle/nk	unknown status	0.125	0.125	0.063

Annotations: All isolates originated from Friedrich-Loeffler-Institut, Institute of Farm Animal Genetics, and some of the isolates were previously published (Kehrenberg 1998; Kehrenberg 2000; Kehrenberg 2001; Kehrenberg 2005).

1) D, suffering from respiratory diseases; **H,** healthy; **nk,** not known.

2) AMP, ampicillin; **CHL,** chloramphenicol; **GEN,** gentamicin; **KAN,** kanamycin; **SPT,** spectinomycin; **SXT,** sulfamethoxazol/trimethoprim; **STR,** streptomycin; **SUL,** sulphonamides; **TET,** tetracycline; **TMP,** trimethoprim.

References

85. Kehrenberg, C.; Catry, B.; Haesebrouck, F.; de Kruif, A.; Schwarz, S. *tet*(L)-mediated tetracycline resistance in bovine *Mannheimia* and *Pasteurella isolates*. J Antimicrob Chemother, **2005**, 56, 403-6. https://doi.org/10.1093/jac/dki210.
86. Kehrenberg, C.; Salmon, S. A.; Watts, J. L.; Schwarz, S. Tetracycline resistance genes in isolates of *Pasteurella multocida*, *Mannheimia haemolytica*, Mannhe*imia glucosida* and *Mannheimia varigena* from bovine and swine respiratory disease: intergeneric spread of the *tet(H)* plasmid pMHT1. *J Antimicrob Chemother*, **2001**, 48, 631-40. https://doi.org/ 10.1093/jac/48.5.631.
87. Kehrenberg, C; Schwarz, S. Identification of a truncated, but functionally active *tet(H)* tetracycline resistance gene in *Pasteurella aerogenes* and *Pasteurella multocida*. FEMS Microbiol Lett, **2000**, 188, 191-5. https://doi.org/10.1111/j.1574-6968.2000.tb09192.x.
88. Kehrenberg, C.; Werckenthin, C.; Schwarz, S. Tn*5706*, a transposon-like element from *Pasteurella multocida* mediating tetracycline resistance. Antimicrob Agents Chemother, **1998**, 42, 2116-8. https://doi.org/10.1128/AAC.42.8.2116.
